# The effect of luseogliflozin on bone microarchitecture in older patients with type 2 diabetes: study protocol for a randomized controlled pilot trial using second-generation, high-resolution, peripheral quantitative computed tomography (HR-pQCT)

**DOI:** 10.1186/s13063-020-04276-4

**Published:** 2020-05-05

**Authors:** Ai Haraguchi, Riyoko Shigeno, Ichiro Horie, Shimpei Morimoto, Ayako Ito, Ko Chiba, Yurika Kawazoe, Shigeki Tashiro, Junya Miyamoto, Shuntaro Sato, Hiroshi Yamamoto, Makoto Osaki, Atsushi Kawakami, Norio Abiru

**Affiliations:** 1 Department of Endocrinology and Metabolism, Division of Advanced Preventive Medical Sciences, Nagasaki University Graduate School of Biomedical Sciences, 1-7-1 Sakamoto, Nagasaki, 852-8501 Japan; 2 Division of Advanced Preventive Medical Sciences, Department of Endocrinology and Metabolism, Nagasaki University Graduate School of Biomedical Sciences, 1-7-1 Sakamoto, Nagasaki, 852-8501 Japan; 3 Innovation Platform and Office for Precision Medicine, Nagasaki University Graduate School of Biomedical Sciences, 1-7-1 Sakamoto, Nagasaki, 852-8501 Japan; 4 Department of Orthopedic Surgery, Nagasaki University Graduate School of Biomedical Sciences, 1-7-1 Sakamoto, Nagasaki, 852-8501 Japan; 5 Clinical Research Center, Nagasaki University Hospital, 1-7-1 Sakamoto, Nagasaki, 852-8501 Japan

**Keywords:** Type 2 diabetes, Luseogliflozin, SGLT2 inhibitor, HR-pQCT, Fracture, Bone, Randomized controlled trial, Pilot

## Abstract

**Background:**

Older patients with type 2 diabetes mellitus (T2DM) have an increased risk of bone fracture independent of their bone mineral density (BMD), which is explained mainly by the deteriorated bone quality in T2DM compared to that in non-diabetic adults. Sodium-glucose co-transporter (SGLT) 2 inhibitors have been studied in several trials in T2DM, and the Canagliflozin Cardiovascular Assessment Study showed an increased fracture risk related to treatment with the SGLT2 inhibitor canagliflozin, although no evidence of increased fracture risk with treatment with other SGLT2 inhibitors has been reported. The mechanism of the difference in the fracture risk between the SGLT2 inhibitors is unknown, but the differences among the SGLT2 inhibitors in the selectivity of SGLT2 against SGLT1 may affect bone metabolism, since among the SGLT2 inhibitors the selectivity of canagliflozin is lowest. We will investigate whether the SGLT2 inhibitor luseogliflozin, which has the higher SGLT2 selectivity, affects bone metabolism by using high-resolution, peripheral quantitative computed tomography (HR-pQCT) which provides direct in vivo morphometric information about the bone microarchitecture.

**Methods/design:**

This is a single-center, randomized, open-label, active-controlled, parallel pilot trial. Eligible participants are older (age ≥ 60 years) individuals with T2DM with HbA1c levels at 7.0–8.9%. A total of 24 participants will be allocated to either the luseogliflozin group (taking luseogliflozin) or the control group (taking metformin) in a 1:1 ratio to compare the groups’ changes in bone microarchitecture of the radius and tibia which are analyzed by HR-pQCT before and at 48 weeks after the administration of each medication. The laboratory data associated with glycemic control and bone metabolism will be collected every 12 weeks during the study. Recruitment began in June 2019.

**Discussion:**

The reason that we use metformin as an active control is to avoid yielding differences in glycemic control between the luseogliflozin and control groups. Besides, metformin is considered to have a neutral effect on bone. This trial should reveal the effect of luseogliflozin on bone metabolism in older patients with T2DM.

**Trial registration:**

The study was registered with the University Hospital Medical Information Network (UMIN000036202) on 1 April 2019 and with the Japan Registry of Clinicla Trials (jRCTs071180061) on 14 March 2019.

## Background

Type 2 diabetes mellitus (T2DM) is well known as a cause of secondary osteoporosis. Individuals with T2DM have shown a 40–70% increase in the risk of hip fracture compared to age- and gender-matched healthy adults [1, 2]. However, the bone mineral density (BMD) of the spine and femur in individuals with T2DM has been reported to be comparable to, or somewhat higher, than that in non-diabetic adults [1, 3]. The cause of the elevated fracture risk in the individuals with T2DM is, thus, suspected to be deteriorated bone quality due to (1) the accumulation of advanced-glycation end-products in the bone collagen fibers and (2) decreased bone turnover as a result of exposure to hyperglycemia [3, 4].

High-resolution, peripheral quantitative computed tomography (HR-pQCT), first developed in 2004, is a three-dimensional imaging technology. HR-pQCT is effective for determining bone quality based on an evaluation of microarchitecture, volumetric BMD, and geometry separately for the cortical and trabecular compartments of the distal radius and tibia. This technique can often detect a fracture risk that is independent of the BMD determined by dual-energy x-ray absorptiometry (DXA) [5–7]. Several studies evaluated the bone microstructure in individuals with T2DM using first-generation HR-pQCT, and the results regarding the deteriorated bone quality were controversial [8–11]. A second-generation HR-pQCT scanner was recently developed with an improved ability to analyze bone microarchitecture; this second-generation HR-pQCT scanner has not yet been used in a study of individuals with T2DM.

Sodium glucose co-transporter 2 (SGLT2) inhibitors have garnered attention as a breakthrough drug for patients with T2DM, as it was revealed that SGLT2 inhibitors have the potential to improve cardiovascular outcomes in T2DM [12–14]. However, there are several concerns about the SGLT2 inhibitors’ effects on bone metabolism regarding the following: (1) stimulation of parathyroid hormone (PTH) and fibroblast growth factor 23 by the increased serum phosphate levels via tubular reabsorption in phosphate, (2) weight loss, and (3) volumetric depletion and fall risk (e.g., orthostatic hypotension and postural dizziness) [15].

The U.S. Food and Drug Administration recently strengthened the warning for the SGLT2 inhibitor canagliflozin related to an increased risk of bone fracture, based on the outcomes in the Canagliflozin Cardiovascular Assessment Study (CANVAS) [16]. In that study, the rate of fracture adverse events (AEs) in the canagliflozin group was significantly increased compared to that of the placebo group (4.0% vs. 2.6%, respectively) [17]. It was also reported that canagliflozin treatment was associated with a small but significant reduction in the BMD of the total hip and with increases in bone biomarkers of formation (osteocalcin) and resorption (collagen type 1 β-carboxytelopeptide) compared to placebo controls [18], which might be explained, at least in part, by canagliflozin-treated patients’ weight loss. In contrast, no evidence of an increased fracture risk has been reported in other cardiovascular outcome trials evaluating the effects of SGLT2 inhibitors in the patients with T2DM [12, 19, 20].

There is no clear explanation for the difference in fracture risk between the CANVAS trial and other trials. It is possible that among the SGLT2 inhibitors, the selectivity of SGLT2 against SGLT1 may affect bone metabolism and the fracture risk, since the selectivity of canagliflozin is the lowest among the SGLT2 inhibitors under clinical development [21]. However, it is not yet known whether the CANVAS trial’s finding of an increased rate of fracture events related to canagliflozin treatment would be reproduced by treatment with other SGLT2 inhibitors, especially those with higher SGLT2 selectivity.

## Methods/design

### Objective

This study will aim to determine whether treatment with the SGLT2 inhibitor luseogliflozin (which has high SGLT2 selectivity) affects bone metabolism by assessing the changes in bone microstructure using second-generation HR-pQCT in older patients with T2DM.

### Study design

**Fig. 1:**
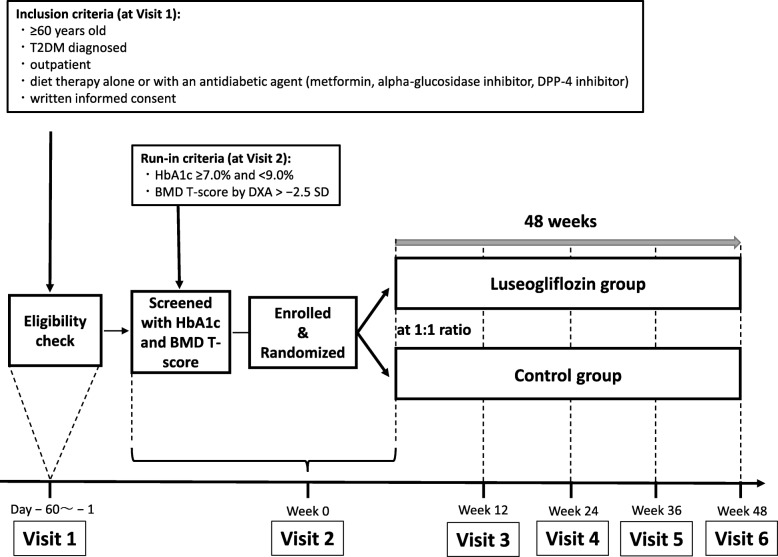
The study design. The eligibility check is conducted and the patient’s informed consent is obtained at visit 1. Only the patients who satisfy the run-in criteria at visit 2 are enrolled in the study; they are randomized at a 1:1 ratio to the luseogliflozin group (administered luseogliflozin 2.5 mg) and the control group (administered metformin 500 mg). The bone microarchitecture of the patients is analyzed before (visit 2) and 48 weeks after the administration of each medication (visit 6). *BMD* bone mineral density, *SD* standard deviation, *T2DM* type 2 diabetes mellitus

This is a pilot trial to assess the effect of SGLT2 inhibitors on bone metabolism. We designed a single-center, randomized, open-label, parallel-group, active-controlled trial for patients with T2DM whose conditions have never been complicated by osteoporosis. We will randomly assigned 24 patients using a 1:1 ratio to the luseogliflozin group (receiving additional treatment with luseogliflozin) or the control group (receiving additional treatment with metformin). The study follow-up duration is 48 weeks. The study will be conducted at Nagasaki University Hospital, Japan. The study design is summarized in Fig. 1.

### Outcomes

The primary outcome measures are changes in the predicted bone strength as determined by second-generation HR-pQCT, evaluated using the parameters of: (1) bone stiffness and (2) the estimated failure load of the radius and tibia of the nondominant body side. For each parameter, any change will be determined based on the difference between the measurement results at baseline (week 0) and week 48.

The study’s secondary outcome measures are as follows: (1) the changes in the structures of cortical bone, trabecular bone, and the bone morphology measured by HR-pQCT as described below in the “Image measurements” section from baseline (week 0) to week 48; (2) the changes in the laboratory data values, including the levels of glycated hemoglobin (HbA1c) from week 0 to weeks 12, 24, 36, and 48; (3) the changes in the areal BMD of the lumbar spine (L1–L4), femoral neck, and distal radius estimated by DXA from week 0 to week 48; (4) the incidence of vertebral fracture or femoral fracture from week 0 to week 48; and (5) changes in the bone metabolic markers from week 0 to week 48.

In addition to the primary and secondary outcomes, we will evaluate adverse effects from both luseogliflozin and metformin (control agent). We will also evaluate the recruitment rate and consent rate.

### Sample size estimation

This is a pilot trial to assess the changes in bone microstructure affected by luseogliflozin treatment compared with metformin treatment, evaluated by HR-pQCT. There is no prior similar study comparing the bone strength before and after the intervention that can be used to estimate the precise optimal sample size. Julious et al. reported that 12 participants per group are needed for a pilot study [22]. The justification for this sample size is based on the rationale concerning feasibility and precision regarding the mean and variance of the primary outcome measures.

### Patients and public involvement statement

There is no patient or public involvement in this trial.

### Participants and recruitment

A total of 24 participants aged ≥ 60 years will be recruited into the study. The enrollment started in June 2019. All participants have been diagnosed with T2DM, and their cases have never been complicated with osteoporosis. Patients fulfilling the inclusion criteria described below will be invited to a screening for their eligibility. The principal investigator and co-investigators recruit the participants among their outpatients, and obtain a written informed consent from the participants. There are no additional consent provisions for the collection and use of participant data and biological specimens in ancillary studies. The recruitment rate and the consent rate will be evaluated at the end of the study.

### Inclusion criteria

As shown in Fig. 1, participants must fulfill the following criteria to be eligible for inclusion at their first visit to Nagasaki University Hospital (visit 1): (1) an individual aged ≥ 60 years; (2) diagnosed with T2DM; (3) an outpatient; (4) treated with diet therapy alone or with orally administered antidiabetic agents including metformin (≤ 1000 mg/day), and/or an alpha-glucosidase inhibitor (α-GI) and/or a dipeptidyl peptidase-4 (DPP-4) inhibitor, whose treatment has not been changed within 6 months before enrollment; and (5) providing written informed consent.

### Run-in criteria

Participants will be included when they also fulfill the following criteria at week 0 (visit 2): (1) the patient’s HbA1c (National Glycohemoglobin Standardization Program: NGSP) level is ≥ 7.0% and < 9.0%; (2) the patient’s T score in both lumbar vertebrae and the femoral neck determined by DXA is > − 2.5 standard deviations (SDs).

### Exclusion criteria

Eligible participants will be excluded if they meet any of the following criteria: (1) have a previous history of treatment with a SGLT2 inhibitor; (2) are complicated by a bone metabolic disorder (i.e., osteomalacia, thyroid dysfunction, hyperparathyroidism, or hypoparathyroidism which requires any medical treatments); (3) have been administered any treatment for osteoporosis within 12 months before enrollment (e.g., a bisphosphonate, vitamin D preparation, vitamin K preparation, calcium preparation, selective estrogen-receptor modulator, antireceptor activator of nuclear-factor kappa-Β ligand antibody (denosumab), anti-sclerostin antibody (romosozumab), or recombinant human PTH (teriparatide)); (4) are being treated with estrogen or testosterone hormone replacement therapy; (5) are complicated by diabetic retinopathy and/or diabetic neuropathy that required therapeutic intervention; (6) have severe renal dysfunction defined as an estimated glomerular filtration rate (eGFR) < 30 mL/min/1.73 m^2^; (7) have severe anemia (Hb < 10 g/dL); (8) are complicated by malignancy; (9) have a hypersensitivity or allergy to metformin and/or luseogliflozin; (10) have an alcohol consumption > 20 g/day; (11) have a smoking habit, including smoking even one cigarette per day, within 12 months before enrollment; (12) are participating in other clinical research within 6 months before enrollment; (13) are complicated by chronic liver disease with ≥ 6 points on the Child-Pugh score; (14) have a body mass index (BMI) < 18.5 kg/m^2^; and (15) are otherwise deemed inappropriate by the study’s principal investigator.

### Withdrawal criteria

Participants will be withdrawn from this trial after randomization if they meet any of the following criteria: (1) are exhibiting an increase in HbA1c level that is ≥ 0.5% of the baseline (week 0) for any two successive visits; (2) have a HbA1c level > 10.0% at any visit; (3) the occurrence of any AE that is ≥ grade 3 of the Common Terminology Criteria for Adverse Events (CTCAE) ver. 4.0; (4) are showing any fracture(s) with the exception of an occult fracture; (5) have requested withdrawal from the study; (6) have requested a change in, or the discontinuation of, the diabetes treatment; (7) it is determined that it is inappropriate for the patient to continue in the study by the investigators because of the progression of an underlying disease or a complication, or the occurrence of an unidentified disease; and (8) it is judged by the investigators that it is inappropriate for the patient to continue participating in the study for any other reason.

### Ethical considerations

This protocol was approved by Clinical Research Review Board of Nagasaki University which is certified by Japan’s Ministry of Health, Labor, and Welfare (approval no. CRB18–0006). The study is registered on the University Hospital Medical Information Network Clinical Trials Registry (UMIN-CTR) as UMIN-CTR no. 000036202. The study is conducted in accordance with the 7th revision of the Declaration of Helsinki 2013 and the Clinical Trials Act enforced in April 2018 in Japan.

### Setting and study timeline

All participants are recruited at Nagasaki University Hospital. Each participant’s information as listed below is collected at the pre-screening for enrollment (visit 1); age, sex, height, comorbid complications, diabetes duration, history of diabetes treatment, family history of diabetes and osteoporosis within the second degree of kinship, and histories of smoking, drinking, and fracture. Body weight, blood pressure, and pulse rate in a sedentary position are measured at every visit of the study (at visits 1–6, and (if withdrawing from the study) at withdrawal). As shown in Table 1, the participants will undergo examinations by spinal x-ray, DXA, and HR-pQCT at week 0 (visit 2) and week 48 (visit 6). When participants need to be withdrawn from the study for any reason, they will undergo HR-pQCT within 4 weeks after discontinuing the study drug. Blood specimens are obtained at every visit except the pre-screening visit (visit 1).

**Table 1:** The schedule of pre-screening, interventions, and assessments

Assessment	Pre-screening	Baseline	Monitoring				Withdrawal
Time	Day 60~ −1	Week 0	Week 12	Week 24	Week 36	Week 48	
Visit	Visit 1	Visit 2	Visit 3	Visit 4	Visit 5	Visit 6	
Informed consent	○	–	–	–	–	–	–
Baseline characterization check	○	–	–	–	–	–	–
Adherence check	–	–	•	•	•	•	•
Concomitant treatments							
Adverse events	–						
Vital signs	○	○	•	•	•	•	•
Laboratory data	–	○	•	•	•	•	•
X-ray	–	○	–	–	–	•	–
DXA (BMD measurement)	–	○	–	–	–	•	–
HR-pQCT	–	○	–	–	–	•	•

○ Will be done before initiating the study drug. Will be done after initiating the study drug

A range of ± 4 weeks is allowed for each visit day

The examination at withdrawal would be carried out within 4 weeks from the date of withdrawal

*BMD* bone mineral density, *DXA* dual-energy x-ray absorptiometry, *HR-pQCT* high-resolution, peripheral quantitative computed tomography

### Image measurements

Frontal and lateral x-ray examinations are performed to detect compression fractures of the thoracic and lumbar vertebrae using a semiquantitative technique [23]. DXA is performed for the assessment of the areal BMD at the lumbar spine (L1, L2, L3, L4, L1–4, L-min), total hip (right total, left total), femoral neck (right neck, left neck), and the distal third of the radius (right radius, left radius) using the DXA system (iDXA, GE Medical Systems, WI, USA). The second-generation HR-pQCT (Xtreme CT II, Scanco Medical, Brüttisellen, Switzerland) scans the radius and tibia of the nondominant body side to evaluate the bone microstructure.

We measure the following parameters by HR-pQCT: (1) bone density, i.e., total volumetric bone mineral density, trabecular volumetric bone mineral density, and cortical volumetric bone mineral density; (2) trabecular bone parameters, i.e., bone volume fraction, trabecular thickness, trabecular number, and trabecular separation; (3) cortical bone parameters, i.e., cortical thickness, cortical porosity, and cortical pore diameter; (4) bone morphology, i.e., total area, trabecular area, cortical area, and periosteal perimeter, and (5) predicted bone strength, i.e., stiffness and failure load.

### Laboratory measurements

At visits 3–5, we measure the patients’ complete blood count and plasma levels of sodium, potassium, chlorine, calcium, phosphate, magnesium, total protein, albumin, total cholesterol, high-density lipoprotein cholesterol, low-density lipoprotein cholesterol, triglyceride, blood urea nitrogen, creatinine, total bilirubin, aspartate aminotransferase, alanine aminotransferase, lactate dehydrogenase, alkaline phosphatase, γ-glutamyl transpeptidase, creatine kinase, uric acid, glucose, and HbA1c. At visit 2 and visit 6 (and at withdrawal if discontinuing participation), in addition to the above laboratory data, we measure the serum (plasma) levels of glycoalbumin, C-peptide, glucagon, glutamic acid decarboxylase antibody, intact PTH, whole PTH, free thyroxine, thyroid-stimulating hormone, adrenocorticotropic hormone, luteinizing hormone, follicle-stimulating hormone, total estradiol, total testosterone, sex hormone-binding globulin, total cortisol, growth hormone, insulin-like growth factor-1, homocysteine, glucagon, tartrate-resistant acid phosphatase 5b, total type I procollagen *N*-terminal propeptide, bone-specific alkaline phosphatase, undercarboxylated osteocalcin, osteocalcin, 25-hydroxyvitamin D, 1,25-dihydroxyviamin D, sclerostin, dickkopf-1, and pentosidine. There are no plans for the collection, laboratory evaluation, and storage of biological specimens for genetic or molecular analysis in the current trial and for future use in ancillary studies.

### Randomization and allocation concealment

A randomized, open-label, parallel pilot trial design has been set up. On the day on which the first administration of the study drug (visit 2) is planned after the eligibility assessment, each participant is sequentially allocated to either the luseogliflozin group or the control group in a 1:1 ratio, using a blocked randomization stratified by sex (female or male). The allocation is conducted by the participant-registration center, and the result is sent to the investigators via facsimile so that the block size and randomization schedule are concealed to the investigators, participants, and those assessing outcomes. As the study design is open-label with only outcome assessors being blinded, unblinding will not occur during the study.

### Interventions

1. Luseogliflozin group: participants allocated to the luseogliflozin group will take orally administered luseogliflozin (Taisho Pharma Co., Tokyo, Japan) at 2.5 mg/day from the beginning of the trial (visit 2) in addition to their previous treatment. The dose of luseogliflozin will be increased to 5 mg from visit 3 unless a participant achieves a HbA1c level < 7% or a decrease in the HbA1c of ≥ 0.5% compared to the previous visit. The maximum dose of luseogliflozin permitted is up to 5 mg/day. If a participant cannot achieve the HbA1c levels described above with 5 mg luseogliflozin, an additional treatment with metformin (Sumitomo Dainippon Pharma Co., Osaka, Japan) will be prescribed at each visit by 500-mg increments up to 1500 mg. The daily dose of metformin will be increased by 250 mg at each visit if the participant already takes ≥ 1500 mg/day. The maximum dose of metformin permitted is 2250 mg/day.

2. Control group (metformin group): participants allocated to the control group will have orally administered metformin 500 mg/day newly administered or added from the beginning of the trial (visit 2) in addition to the previous treatment. The additional metformin will be administered at each visit by 500-mg increments unless the participant achieves a HbA1c level < 7% or a decrease in HbA1c ≥ 0.5% compared to the previous visit. The daily dose of metformin will be increased by a 250-mg increment at each visit if the participant already takes ≥ 1500 mg/day. The maximum dose of metformin permitted is 2250 mg/day.

### Adverse events (AEs)

All adverse events (AEs) that occur during the trial will be recorded on a case report form and reviewed as part of the central data monitoring. Investigators explain AEs to the participants and offer appropriate care. If serious AEs, such as death, a life-threatening condition, hospitalization, sequelae, disability, and congenital illness occur during the trial, the principal investigator will report the AE to the Minister of Health, Labor, and Welfare (Japan) and the certified review board within 15 days.

### Data collection, data management and monitoring

All the data will be collected using the research electronic data capture (REDCap) system by the authorized investigators. Once the data are checked, they will be fixed by the Trial Steering Committee (TSC). The TSC provides overall supervision for the trial on behalf of the sponsor and funder and ensures that the trial is conducted according to the rigorous standards set out in the guideline of the Clinical Trials Act. The meeting of the TSC will be held every month over the course of the trial to oversee its conduct and progress. Clinical Porter Inc. (Tokyo, Japan), a site management organization (SMO) specializes in clinical trial management which is independent from the sponsor and has no competing interest, will monitor the overall conduct of the trial, safeguarding the interests of the trial participants and assessing the safety and efficacy of the intervention. The authorized person of the SMO visits before and every year after starting the trial to review protocol compliance, conduct source-data verification, assess laboratory procedures, and ensure that the study is being conducted in accordance with the protocol requirements. Because the risk of intervention in the trial is considered to be extremely low, a regular audit is not conducted in the trial unless serious concerns about the outcomes or/and serious AEs arise. Once a serious event has occurred, the authorized persons of the Clinical Research Support Center of Ryukyu University Hospital, which is independent from the investigators and the sponsor; will make a site visit to audit the trial conduct. The sponsor is responsible for all aspects of local organization including identifying potential recruits and taking consent.

### Statistical analyses

As stated above in the “Objectives” section, the primary outcome of this study is the extent of luseogliflozin’s effects on bone-quality change. Hence, the primary data analysis will be conducted using the set of participants with sufficient exposure to the allocated drugs. For this reason, we will use a per-protocol set (PPS), the definition of which includes the adherence ratio, as the target population in the primary data analysis. The same analyses will be conducted on a full analysis set (FAS) modified FAS (mFAS) to provide supplemental information about the influence of selecting PPS subjects on the results.

As the primary and the secondary data analyses, the adjusted mean and the 95% confidence intervals (95%CIs) of the bone-deterioration effect of the treatment group will be estimated as regression coefficient and its standard error, for the respective primary and secondary outcome measures, under a linear regression model with the following two covariates: the baseline measurement of the respective measures and the stratification factor in the randomization (i.e., sex). This study is set up as a pilot study, but we will test the null hypotheses that “the mean of the bone-quality deterioration effect of the treatment group is 0” by determining whether the 95%CIs contain the null hypotheses.

The respective analysis sets are defined as follows. The intention-to-treat (ITT) population is defined as all participants registered for this trial; the safety analysis set (SAS) population is defined as the participants in the ITT population with at least one administration of luseogliflozin/metformin. The full analysis set (FAS) population is defined as the participants in the SAS for whom data about the predicted bone strength after the administration of luseogliflozin/metformin at one or more scheduled visits are available. The PPS is defined as the participants in the FAS with the primary outcome measure. Other criteria for the PPS will be determined before the database lock of this study. We defined the modified FAS (mFAS), the definition of which is a relaxed version of PPS by allowing missingness of the data of predicted bone strength at week 48 only if the data of the items at withdrawal from this study are obtained.

As a safety analysis, tabular summaries of AE incidence among subjects in a SAS will be created. All hypothesis testing will be conducted at the significance level of 0.05 (two-sided). The data collected will be summarized using the arithmetic mean, the standard deviation, and the quantiles. All statistical analyses are conducted under the computing environment R (R Foundation for Statistical Computing, Vienna, Austria).

## Discussion

The main purpose of this study is to determine whether treatment with the SGLT2 inhibitor luseogliflozin affects bone metabolism in older patients with T2DM by using second-generation HR-pQCT.

For the evaluation of this effect of luseogliflozin, we use metformin as an active control drug for the following three reasons: (1) we should avoid yielding differences in glycemic control between the luseogliflozin and control groups. If we use a placebo control, the effect of luseogliflozin on bone may be confounded by the improvement of glycemic control and (2) metformin is reported to have a neutral effect or at least a not-negative effect on bone [24, 25]. We can avoid overestimating the efficacy of luseogliflozin for bone metabolism by using metformin as a control drug; (3) metformin is the most commonly used antidiabetic agent worldwide and is recommended by most of the relevant guidelines as a first-line therapy for T2DM [14, 26, 27].

Participants treated with an α-GI and/or a DPP-4 inhibitor in addition to metformin can also be enrolled in the study. There is no report demonstrating an increased risk of fracture associated with α-GIs and DPP-4 inhibitors [25, 28]. In addition, those drugs are used widely in Japan for older patients with T2DM.

It is recognized that bone strength is determined by bone mass, morphology, and microstructure. In a clinical setting, an individual’s bone mass is generally represented as the BMD assessed by DXA [7]. We will identify the precise changes in bone strength brought about by 1-year treatment with luseogliflozin by using second-generation HR-pQCT, which can detect the changes of bone morphology and microstructure. Sundh et al. reported that increased cortical porosity determined by HR-pQCT is associated with hip fracture in postmenopausal women, independent of areal BMD in the femoral neck determined by DXA even after adjusting for other fracture risk factors [29]. Samelson et al. reported that the trabecular and cortical bone structure evaluated by HR-pQCT can predict a future risk of femoral fracture, which is independent of the estimated fracture risk provided by the fracture-risk assessment tool (FRAX) [7].

The mega trial CANVAS demonstrated that canagliflozin treatment increased fracture incidents in patients with T2DM compared to those administered a placebo [17]. The increased fracture AEs might to a certain degree be caused by an increased frequency of falls in that patient population, which may have developed secondary to volume contraction or a reduction in blood pressure [17]. The increase in bone fractures observed in the CANVAS trial was not observed in the other CANVAS-like trials regarding the efficacy of canagliflozin, i.e., the CANVAS-Renal trial (CANVAS-R) [13] and eight randomized controlled studies (non-CANVAS studies) [17]. There is no clear explanation for the differences in fracture risk between CANVAS and the nine studies including the CANVAS-R and eight non-CANVAS studies. There is no evidence of an increased fracture risk associated with the other SGLT2 inhibitors with higher selectivity of SGLT2 against SGLT1 compared to that of canagliflozin [12, 19, 20]. It is unclear whether fracture risk is a class effect of the SGLT2 inhibitors.

There are some limitations in the trial. First, the sample size is small (*n* = 24). Second, the follow-up period of the trial may be insufficient to lead to a definitive conclusion regarding the bone effect of luseogliflozin. Third, we cannot deny that the other antidiabetic agents concomitantly used during the trial will influence the participants’ bone metabolism. Despite these limitations, our research also has strengths. There has been no study assessing the effect of antidiabetic drugs on the bone microstructure using HR-pQCT in patients with T2DM. We will be able to elucidate the effect of a SGLT2 inhibitor on bone metabolism by using second-generation HR-pQCT, which provides direct in vivo morphometric information about the bone microarchitecture with a three-dimensional morphological technique.

The main goal of treatment for adolescent or middle-aged patients with T2DM is to prevent cardiovascular disease and microvascular complications such as diabetic retinopathy. However, maintaining the patients’ quality of life, while preventing fractures, is also required when we manage hyperglycemia in fragile older patients with T2DM. It is an important task for clinicians to clarify whether the adverse effects of SGLT2 inhibitors on bone develop in older T2DM patients with a higher fracture risk.

### Trial status

The recruitment is being conducted from June 2019 to December 2020. The current version of this protocol is version 5.0 which was updated on 19 July 2019.

## Supplementary information

**Additional file 1.** Standard Protocol Items: Recommendations for Interventional Trials (SPIRIT) 2013 Checklist: recommended items to address in a clinical trial protocol and related documents*.

## Data Availability

Not applicable

## Abbreviations

T2DM: Type 2 diabetes mellitus
BMD: Bone mineral density
SGLT2: Sodium-glucose co-transporter 2
HR-pQCT: High-resolution, peripheral quantitative computed tomography
DXA: Dual-energy x-ray absorptiometry
CANVAS: The Canagliflozin Cardiovascular Assessment Study
CANVAS-R: CANVAS-Renal trial
